# Continuous chronic musculoskeletal pain and its associated sociodemographic, psychosocial, and behavioral factors among adolescents living in a socially vulnerable urban setting in northeastern Brazil

**DOI:** 10.3389/fpain.2026.2009595

**Published:** 2026-10-30

**Authors:** Francisco Valter Miranda Silva, Ana Paula Vasconcellos Abdon, Alex Silva Ribeiro, Virna Ribeiro Feitosa Cestari, Rhanna Emanuela Fontenele Lima de Carvalho, Saiwori De Jesus Silva Bezerra dos Anjos, Ana Valeska Siebra e Silva

**Affiliations:** 1 Graduate Program in Clinical Care in Nursing and Health, State University of Ceará, Fortaleza, Ceará, Brazil; 2 Graduate Program in Collective Health, University of Fortaleza, Fortaleza, Ceará, Brazil; 3 Faculty of Sport Science and Physical Education, University of Coimbra, FCDEF, Coimbra, Portugal

**Keywords:** adolescents, anxiety, chronic musculoskeletal pain, health equity, pediatric pain, sleep quality, smartphone use, social vulnerability

## Abstract

**Background:**

Chronic musculoskeletal pain is common during adolescence, yet evidence from socially disadvantaged populations in low- and middle-income countries remains limited. This study investigated the occurrence of continuous chronic musculoskeletal pain and its associated sociodemographic, psychosocial, and behavioral factors among adolescents living in a socially vulnerable urban setting in northeastern Brazil.

**Methods:**

An analytical cross-sectional study was conducted with 296 adolescents aged 14–18 years from five public secondary schools in northeastern Brazil. The study area was characterized by socioeconomic deprivation, a Human Development Index below 0.499, and high levels of violence. Musculoskeletal pain was assessed using the validated *Presence and Impact of Pain in Kids* questionnaire, with continuous pain lasting more than three months considered the outcome. Anxiety and depressive symptoms, sleep quality, physical activity, school bullying, family violence, self-rated health, healthcare provision, and daily smartphone use were assessed using standardized instruments and structured questionnaires. Bivariate analyses and multivariable binary logistic regression were performed to estimate crude and adjusted odds ratios.

**Results:**

31.4% adolescents reported continuous chronic musculoskeletal pain. In the final adjusted model, poor sleep quality was associated with higher odds of pain (aOR=2.49; 95% CI: 1.04–5.97; *p* = 0.041), as were anxiety symptoms (aOR=1.93; 95% CI: 1.02–3.63; *p* = 0.042). Each additional hour of daily smartphone use was associated with an 8.8% increase in the odds of pain (aOR=1.09; 95% CI: 1.02–1.16; *p* = 0.011).

**Conclusion:**

Continuous chronic musculoskeletal pain was reported by nearly one-third of adolescents attending public schools in a socially vulnerable urban setting. Its associations with anxiety, poor sleep quality, and longer smartphone use reinforce the multidimensional nature of adolescent pain. These findings expand evidence from an underrepresented Brazilian population and support context-sensitive approaches integrating pain, mental health, sleep, and everyday behaviors within school and primary healthcare settings.

## 1 Introduction

Chronic pain in childhood and adolescence is a significant public health concern, with consequences for physical functioning, mental health, social participation, academic performance, and quality of life. Generally defined as pain that persists or recurs for more than three months, chronic pain is common in pediatric populations. A systematic review and meta-analysis of 119 studies, including more than one million children and adolescents from 70 countries, estimated an overall chronic pain prevalence of 20.8% and identified headache and musculoskeletal pain as the most prevalent pain categories (1). Beyond its high prevalence, persistent pain in early life may affect health and functioning into adulthood (2).

Despite advances in pediatric pain epidemiology, scientific knowledge remains unevenly distributed across geographical regions and populations. A systematic review focusing on low- and middle-income countries identified substantial gaps and marked heterogeneity in the available evidence on pediatric chronic pain, underscoring the limited representation of these settings in the international literature (3). A more recent systematic review of factors associated with and predictive of musculoskeletal pain in adolescence also emphasized the need for research involving diverse populations and socioeconomically disadvantaged settings to improve the generalizability of existing evidence (4). This evidence gap matters because global estimates may obscure differences tied to the social, economic, cultural, and healthcare circumstances in which children and adolescents live.

In Brazil, recent evidence indicates that musculoskeletal pain is also an important health concern among children and adolescents. A study involving 2,688 schoolchildren reported a prevalence of 27.1% for disabling musculoskeletal pain during the preceding month, with pain most frequently affecting the back and lower limbs (5). Although differences in outcome definitions and recall periods preclude direct comparisons between studies, these findings highlight the substantial burden of musculoskeletal pain in Brazilian pediatric populations. Because much of the available literature comes from better-resourced settings, pooled global estimates may not adequately reflect variation in chronic pain burden across populations with different socioeconomic conditions, cultural contexts, and access to healthcare (1). Nevertheless, evidence specifically describing chronic musculoskeletal pain among adolescents living in socioeconomically disadvantaged Brazilian urban settings remains limited.

Understanding pediatric pain requires recognition that its occurrence and consequences are not determined exclusively by individual biological factors. Social determinants related to economic stability, school environments, neighborhood characteristics, community circumstances, and access to healthcare may coexist with and influence the experience of pain (6). In a sample of 30,287 children and adolescents, Chen et al. (6) found higher odds of chronic pain among those exposed to food insecurity, parental unemployment, lower school safety, limited access to healthcare, bullying, and adverse community experiences. These findings underscore the relevance of an equity-oriented approach, particularly among populations living in socially disadvantaged areas. However, contextual indicators of vulnerability should be interpreted as characteristics of the social environments in which young people live, rather than as direct measures of individual socioeconomic circumstances or evidence of a causal relationship with pain (7).

Within this context, psychological and behavioral factors also contribute to the multidimensional nature of pain during adolescence. A meta-analysis of 79 studies demonstrated a high prevalence of anxiety and depression among young people with chronic pain, alongside greater psychological symptom severity compared with their peers without pain (8). Sleep disturbances have also been associated with musculoskeletal pain and related disability in adolescents (9). At the same time, changes in digital behavior have raised new questions about musculoskeletal health. A recent study of Colombian adolescents found associations between dimensions of mobile phone dependence and musculoskeletal pain, particularly neck pain (10). These factors should not be understood in isolation or as deterministic influences, but rather as potentially interrelated components of the pain experience, shaped by individual characteristics and the broader social context.

Evidence on continuous chronic musculoskeletal pain and its psychosocial, sleep-related, and digital-behavior correlates remains limited in low- and middle-income countries and socioeconomically disadvantaged settings (3, 4). Evidence from these populations may improve the representation of underserved groups in pediatric pain research and inform context-sensitive approaches to assessment and care. Therefore, this study aimed to investigate the occurrence of continuous chronic musculoskeletal pain and its associated sociodemographic, psychosocial, and behavioral factors among adolescents living in a socially vulnerable urban setting in north-eastern Brazil.

## 2 Methods

An analytical cross-sectional study was conducted as part of a larger research project entitled “Biopsychosocial Factors and the Occurrence of Chronic Musculoskeletal Pain among Adolescents Attending Public Schools in a Remote Area of Fortaleza”. The study was conducted in five public secondary schools in socially vulnerable territories within Regional I of Fortaleza, Ceará, northeastern Brazil.

The selected area is characterized by a concentration of neighborhoods experiencing substantial socioeconomic deprivation and exposure to violence, including areas with higher poverty rates, a Human Development Index (HDI) below 0.499, and high levels of crime and violence, reflected in a homicide rate of 45.3 per 100,000 inhabitants (11). Schools were selected using two-stage stratified sampling, with the five educational territories of the administrative region serving as strata. Initially, one school was randomly selected from each territory. Subsequently, classes from all three secondary-school grades were randomly selected to achieve a proportional distribution of participants. When the required number of participants was not reached, the team randomly selected additional classes until it reached the target sample size for each stratum. A previously trained research team collected data in person between August and December 2025 in classrooms or private spaces provided by the schools, under the supervision of the principal investigator.

Adolescents aged 14–18 years who were regularly enrolled in and attending the selected schools were eligible for inclusion. Those reporting musculoskeletal pain secondary to trauma or fracture, neurological, oncological, or degenerative diseases, or other specific conditions that could explain their pain were excluded. Pregnant adolescents and participants with visual or hearing impairments that prevented them from completing the study instruments were also excluded. The minimum required sample size was 293 participants, calculated using a formula (***n*** **=** [1*Np(1-p)]/ [(d^2^/Z21-*α*/2*(N-1)+p*(1-p)]) for cross-sectional studies for a finite population through OpenEpi® software (12) a population of 7,664 students (13), an expected musculoskeletal pain prevalence of 27.1% (5), a 5% margin of error, and a 95% confidence level. To account for an anticipated 10% non-response, the recruitment target was increased to 326 participants and rounded upward to 330 for field recruitment. The final analytical sample comprised 296 adolescents with complete eligible data, exceeding the minimum required analytical sample.

Before data collection, parents or legal guardians provided written informed consent, and adolescents provided written assent. The principal investigator and trained research team verbally explained the risks and benefits of participation, as described in the written consent and assent forms. The study ensured voluntary participation, confidentiality, and anonymity. The study was approved by the Research Ethics Committee of the State University of Ceará (approval no. 5.526.758).

The presence of musculoskeletal pain was assessed using the Presence and Impact of Pain in Kids (PIP-Kids) questionnaire, which has been translated and validated for Brazilian children and adolescents. The instrument comprises 10 items in two sections: items 1–5 assess the presence of musculoskeletal pain, while items 6–10 assess its impact. The Brazilian-Portuguese version of the PIP-Kids was evaluated in 656 children and adolescents. Confirmatory factor analysis supported its two-domain structure (presence and impact of pain), test–retest reliability assessed using kappa coefficients ranged from 0.20 to 0.68, percentage agreement ranged from 60.2% to 92.0%, and 80.5% of prespecified hypotheses for construct validity were confirmed (14). In the present study, continuous chronic musculoskeletal pain was operationally defined as an affirmative response to item 5, indicating the presence of continuous pain lasting longer than three months. The descriptive analysis also included items on pain treatment, medication use, and school absence due to pain. This temporal criterion is consistent with the definition of chronic pain as pain that persists or recurs for more than three months (15).

Sociodemographic characteristics were collected using a structured questionnaire based on the Brazilian National Health Survey conducted by the Brazilian Institute of Geography and Statistics (IBGE) (16). Variables included sex, age, self-reported skin color, self-rated health, type of healthcare provision, and daily smartphone use (hours). Smartphone use was self-reported by participants based on usage information available on their own devices. In addition to daily smartphone use in hours, participants reported the purposes for which they used their smartphones, including phone calls, gaming, watching films or videos, social networking, WhatsApp messaging, and studying. Multiple responses were permitted. Exposure to family violence and school bullying was assessed using specific questions, with both variables classified as absent or present.

Physical activity levels were assessed using the short version of the International Physical Activity Questionnaire (IPAQ). Participants were classified into three categories according to weekly physical activity: active (>150 min/week), insufficiently active (10–150 min/week), and sedentary (<10 min/week) (17). Sleep quality was assessed using the Pittsburgh Sleep Quality Index (PSQI), which has been translated and validated for Brazilian Portuguese and assesses sleep quality and disturbances over the preceding 7 and 30 days; the validation study reported good internal consistency for the PSQI, with a Cronbach's alpha of 0.82. The instrument comprises 19 self-rated questions organized into seven components. Each component is scored from 0 to 3, yielding a total score ranging from 0 to 21. A global score of ≤5 was used to indicate good sleep quality, whereas scores >5 indicated poor sleep quality (18).

Symptoms of anxiety and depression were assessed using the 21-item Depression Anxiety and Stress Scale (DASS-21), which has been translated and adapted into Brazilian Portuguese and validated for Brazilian adolescents. The DASS-21 subscales demonstrated adequate internal consistency (0.83–0.90), and confirmatory factor analysis supported the original three-factor structure comprising depression, anxiety, and stress (19). The depression and anxiety subscales were used. For the analyses, participants scoring within the normal range were classified as not presenting symptoms, whereas those classified as having mild symptoms or greater severity were considered to present symptoms.

To minimize potential selection bias, schools and classes were randomly selected using a territory-stratified sampling approach. Data were obtained using standardized instruments and self-administered questionnaires completed under the supervision of a previously trained research team. In the multiple logistic regression models, potential confounding was addressed by including theoretically relevant variables and those with *p*-values below 0.20 in the bivariate analysis.

Statistical analyses were performed using IBM® SPSS Statistics, version 23.0. Initially, the distribution of numerical variables was assessed using the Kolmogorov–Smirnov normality test. Normally distributed variables were summarised as means and standard deviations, whereas categorical variables were presented as absolute and relative frequencies. In the bivariate analysis, Pearson's chi-squared test was used to examine associations between continuous chronic musculoskeletal pain and the investigated variables. Crude odds ratios (ORs) and their corresponding 95% confidence intervals (95% CIs) were estimated using univariable binary logistic regression models. For the multivariable analysis, variables with *p*-values below 0.20 in the bivariate analysis, as well as those considered relevant on theoretical grounds, were included in multiple logistic regression models. Results were expressed as adjusted ORs and their corresponding 95% CIs, obtained by exponentiating the regression coefficients (β). Model fit and performance were assessed using the Hosmer–Lemeshow goodness-of-fit test, Nagelkerke's pseudo-R^2^, and overall classification accuracy. A significance level of 5% (*p* < 0.05) was adopted for all analyses.

## 3 Results

The study included 296 adolescents (mean age 16.2 ± 1.3 years), of whom 48.6% were female. Brown (31.4%) and White (29.7%) were the most frequently reported skin color categories. Most participants reported using public healthcare services (65.2%), while 7.8% engaged in paid employment. Poor self-rated health was reported by 40.9% of participants. Anxiety and depressive symptoms were identified in 51.7% and 34.8% of adolescents, respectively, while 55.7% reported exposure to family violence and 39.2% reported school bullying. Regarding behavioral and sleep-related characteristics, 74.7% were classified as physically active, 81.8% reported poor sleep quality, and mean daily smartphone use was 7.3 ± 3.8 h. Overall, 93 adolescents (31.4%) reported continuous chronic musculoskeletal pain. Among adolescents with continuous chronic musculoskeletal pain (*n* = 93), 21 (22.6%) reported seeking health professional advice or treatment for pain, whereas 65 (69.9%) reported taking medication to relieve pain. Regarding smartphone use patterns, social networking was the most frequently reported activity (82.8%), followed by WhatsApp messaging (54.7%), watching films or videos (39.2%), studying (35.8%), gaming (31.8%), and phone calls (29.4%) (Table 1). Because participants could report more than one purpose of smartphone use, these categories were not mutually exclusive.

**Table 1:** Description of the variables investigated (*n* = 296).

Variables	*n*	%	Mean ± SD
Sociodemographic characteristics			
Age (years)	—	—	16.2 ± 1.3
Sex			
Male	152	51.4	—
Female	144	48.6	—
Self-reported skin color			
Brown	93	31.4	—
White	88	29.7	—
Black	43	14.5	—
Yellow	67	22.6	—
Indigenous	5	1.7	—
Paid employment			
No	273	92.2	—
Yes	23	7.8	—
Public healthcare provision			
No (private)	103	34.8	—
Yes (public)	193	65.2	—
Psychosocial characteristics			
Poor self-rated health			
No	175	59.1	—
Yes	121	40.9	—
Depressive symptoms			
No	193	65.2	—
Yes	103	34.8	—
Anxiety symptoms			
No	143	48.3	—
Yes	153	51.7	—
Exposure to family violence			
No	131	44.3	—
Yes	165	55.7	—
Exposure to school bullying			
No	180	60.8	—
Yes	116	39.2	—
Behavioral and sleep-related characteristics			
Physical activity level			
Active	221	74.7	—
Insufficiently active	23	7.8	—
Sedentary	52	17.6	—
Sleep quality			
Good	54	18.2	—
Bad	242	81.8	—
Daily smartphone use (hours/day)	—	—	7.3 ± 3.8
Purposes of smartphone use^a^			
Social networking (yes)	245	82.8	—
WhatsApp messaging (yes)	162	54.7	—
Watching films/videos (yes)	116	39.2	—
Studying (yes)	106	35.8	—
Gaming (yes)	94	31.8	—
Phone calls (yes)	87	29.4	—
Continuous chronic musculoskeletal pain			
No	203	68.6	—
Yes	93	31.4	—

n, absolute frequency; %, ercentage; SD, standard deviation.

a Smartphone-use categories were not mutually exclusive; multiple responses were permitted.

In the bivariate analysis, continuous chronic musculoskeletal pain was associated with female sex (OR = 1.98; 95% CI: 1.20–3.26; *p* = .007), anxiety symptoms (OR = 2.83; 95% CI: 1.68–4.75; *p* < .001), depressive symptoms (OR = 2.19; 95% CI: 1.32–3.64; *p* = .002), and exposure to school bullying (OR = 1.74; 95% CI: 1.06–2.87; *p* = .028). Poor sleep quality (OR = 3.70; 95% CI: 1.60–8.54; *p* = .001) and poor self-rated health (OR = 2.03; 95% CI: 1.23–3.34; *p* = .005) were also associated with the outcome. Public healthcare provision was associated with lower odds of continuous pain compared with private healthcare provision (OR = 0.56; 95% CI: 0.34–0.93; *p* = .223). Mean daily smartphone use was higher among adolescents with continuous pain (8.43 ± 4.08 h/day) than among those without continuous pain (7.02 ± 4.06 h/day; *p* = .006). No statistically significant differences were observed for age, family violence, or physical activity level (Table 2).

**Table 2:** Bivariate analysis of factors associated with continuous chronic musculoskeletal pain among adolescents (*n* = 296).

Variables	Without continuous pain (*n* = 203)	With continuous pain (*n* = 93)	OR (95% CI)	*p*-value
Sociodemographic				
Sex				
Male	115 (56.7)	37 (39.8)	1.00	—
Female	88 (43.3)	56 (60.2)	1.98 (1.20–3.26)	.007*
Age (years), mean ± SD	16.28 ± 1.02	16.10 ± 1.91	—	.282
Psychosocial				
Anxiety symptoms				
No	114 (56.2)	29 (31.2)	1.00	—
Yes	89 (43.8)	64 (68.8)	2.83 (1.68–4.75)	<.001*
Depressive symptoms				
No	144 (70.9)	49 (52.7)	1.00	—
Yes	59 (29.1)	44 (47.3)	2.19 (1.32–3.64)	.002*
School bullying				
No	132 (65.0)	48 (51.6)	1.00	—
Yes	71 (35.0)	45 (48.4)	1.74 (1.06–2.87)	.028*
Family violence				
No	91 (44.8)	40 (43.0)	1.00	—
Yes	112 (55.2)	53 (57.0)	1.08 (0.66–1.77)	.770
Behavioural				
Physical activity level				.382
Active	147 (72.4)	74 (79.6)	1.00	
Insufficiently active	18 (8.9)	5 (5.4)	0.55 (0.20–1.54)	—
Sedentary	38 (18.7)	14 (15.1)	0.73 (0.37–1.44)	
Daily smartphone use (hours)	7.02 ± 4.06	8.43 ± 4.08	—	.006*
Sleep quality				
Good	47 (23.2)	7 (7.5)	1.00	—
Poor	156 (76.8)	86 (92.5)	3.70 (1.60–8.54)	.001*
Self-rated health				
Good	131 (64.5)	44 (47.3)	1.00	—
Bad	72 (35.5)	49 (52.7)	2.03 (1.23–3.34)	.005*
Type of healthcare provision				
Private	62 (30.5)	41 (44.1)	1.00	—
Public	141 (69.5)	52 (55.9)	0.56 (0.34–0.93)	.223

n, absolute frequency; %, percentage; OR, odds ratio. 95%CI, 95% Confidence Interval; *p < .05, Chi-square test.

In the final multivariable logistic regression model, poor sleep quality (aOR = 2.49; 95% CI: 1.04–5.97; *p* = .041), anxiety symptoms (aOR = 1.93; 95% CI: 1.02–3.63; *p* = .042), and longer daily smartphone use (aOR = 1.09 per additional hour; 95% CI: 1.02–1.16; *p* = .011) remained independently associated with higher odds of continuous chronic musculoskeletal pain. In contrast, female sex, depressive symptoms, school bullying, and poor self-rated health were not significantly associated with the outcome after adjustment (all *p* > .05). These findings are detailed in Table 3 and illustrated in Figure 1.

**Table 3:** Multivariable logistic regression analysis of factors associated with continuous chronic musculoskeletal pain among adolescents (*n* = 296).

Variable	Adjusted OR (95% CI)	*p*
Sociodemographic factors		
Female sex	1.38 (0.78–2.42)	.268
Psychosocial factors		
Anxiety symptoms	1.93 (1.02–3.63)	.042*
Depressive symptoms	1.13 (0.62–2.07)	.690
School bullying	1.46 (0.85–2.50)	.168
Behavioural factors		
Daily smartphone use, h/day	1.09 (1.02–1.16)	.011*
Health-related factors		
Poor sleep quality	2.49 (1.04–5.97)	.041*
Poor self-rated health	1.39 (0.80–2.41)	.245

OR, odds ratio; 95%CI, 95% confidence interval; *p < 0.05, logistic regression. Model Quality: The final multivariable model was statistically significant (Omnibus *χ*^2^(7) = 35.97, *p* < .001) and showed adequate calibration (Hosmer–Lemeshow *p* = .944). Nagelkerke's R^2^ was.161, indicating modest explanatory capacity. At the prespecified 0.50 classification threshold, overall accuracy was 69.3%, with a sensitivity of 22.6% and specificity of 90.6%. Accordingly, the model was interpreted as an explanatory model of associations rather than as a clinical prediction tool.

**Figure 1:**
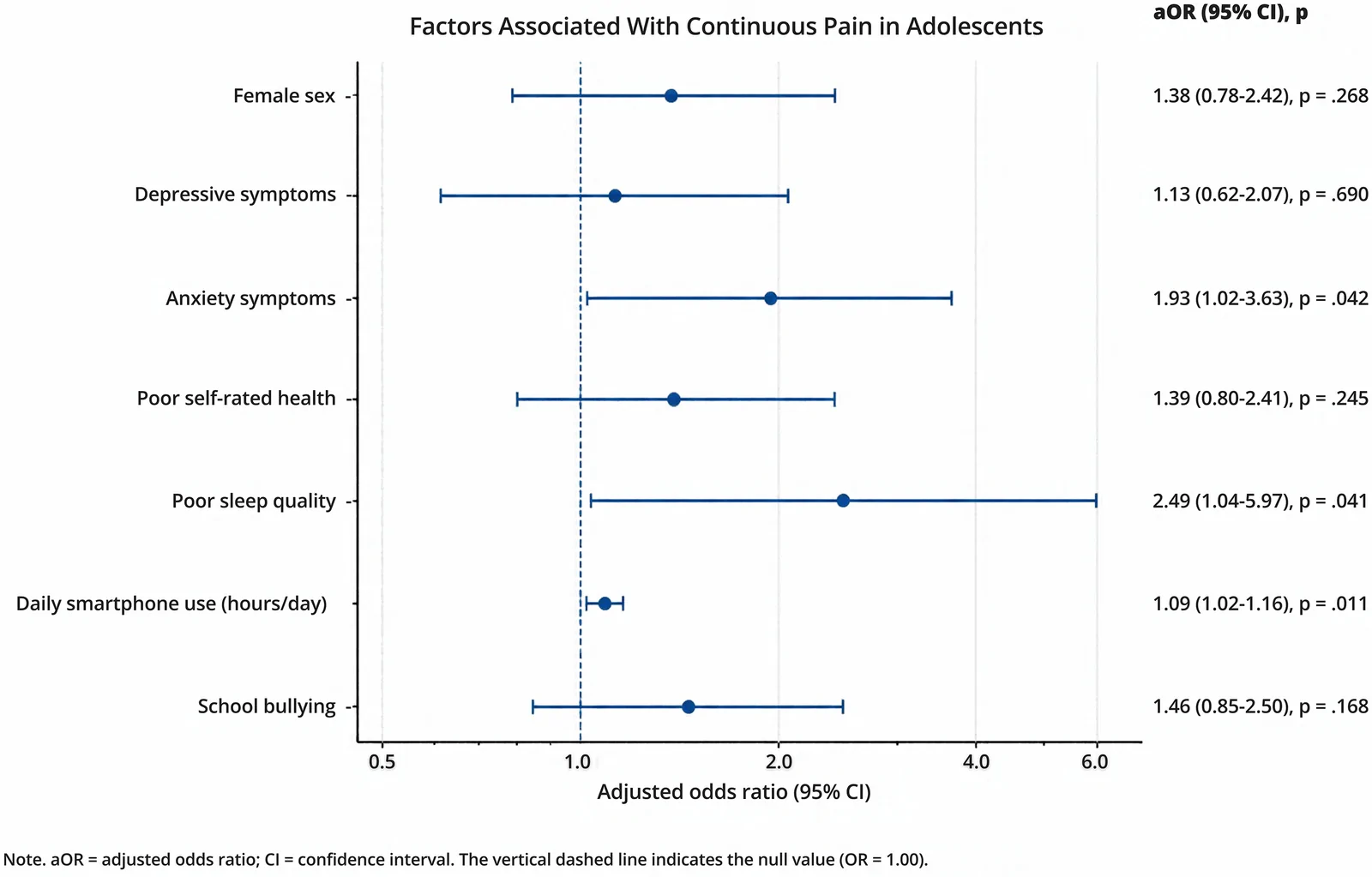
Adjusted odds ratios and 95% confidence intervals for variables included in the final multivariable logistic regression model of continuous chronic musculoskeletal pain among. aOR, djusted odds ratio; CI, confidence interval. The vertical dashed line indicates the null value (aOR = 1.00).

## 4 Discussion

This study aimed to estimate the occurrence of continuous chronic musculoskeletal pain and examine its sociodemographic, psychosocial, and behavioral correlates among adolescents living in a socially vulnerable urban setting in north-eastern Brazil. Nearly one-third of participants reported continuous pain lasting more than three months, and the adjusted analysis identified poor sleep quality, anxiety symptoms, and longer daily smartphone use as the principal correlates of the outcome. This proportion was higher than the pooled global prevalence of chronic pain (20.8%) reported by Chambers et al. (1), as well as the 9% estimate for chronic musculoskeletal pain in low- and middle-income countries (3).

Country-specific studies from other low- and middle-income settings further illustrate the heterogeneity of musculoskeletal pain estimates. In India, Oka et al. (20) reported nonspecific back pain in 38.5% of 1,225 adolescents attending urban and rural schools, with a higher prevalence in urban than rural students (43.4% vs. 33.2%). In Nigeria, the 12-month prevalence of low back pain among secondary-school students was 43.8% (21). Similarly, a recent study of Colombian adolescents reported musculoskeletal pain in 56.3% of participants over the previous six months, with the upper back and neck among the most frequently affected regions (22). In Brazil, Hatakeyama et al. (5) identified a prevalence of 27.1% for disabling musculoskeletal pain among schoolchildren, including participants from Ceará. These estimates are not directly comparable to the present outcome because of differences in anatomical scope, pain definitions, and recall periods, but they reinforce the need for context-specific evidence on pediatric pain.

Pain-related management also warrants consideration. Among adolescents with continuous chronic musculoskeletal pain, medication use was more frequently reported than seeking professional advice or treatment. Physician consultation and medication use represent distinct dimensions of healthcare utilisation among young people with chronic pain and may be influenced by different clinical and contextual factors (20). However, these were separate questionnaire items, and the present study did not determine whether medications were prescribed, recommended by a health professional, obtained over the counter, or self-administered. Therefore, this difference should not be interpreted as evidence of self-medication or lack of access to professional care.

The territorial context is particularly relevant to interpreting these findings. Participants attended schools in a district characterized by socioeconomic deprivation, neighborhood-level Human Development Index values below 0.499, and high levels of violence (11). Previous studies have identified associations between pediatric chronic pain and social determinants, including food insecurity, neighborhood safety, healthcare access, and adverse community experiences (6, 23). Nevertheless, the present study did not assess individual socioeconomic circumstances or compare populations across different levels of territorial vulnerability. Its contribution therefore lies in documenting chronic pain within a disadvantaged urban setting; the study was not designed to estimate the effect of territorial vulnerability on pain.

Anxiety symptoms were independently associated with chronic musculoskeletal pain, consistent with evidence demonstrating a substantial burden of psychological symptoms among young people with chronic pain. Dudeney et al. (24) reported a high prevalence of anxiety among young people with chronic pain, while Harte et al. (8) identified associations between anxiety and greater pain intensity, disability, pain interference, and poorer quality of life. Anxiety may contribute to pain-related distress, whereas persistent pain may also exacerbate psychological symptoms. However, the cross-sectional design does not allow us to determine the direction of this relationship. These findings reinforce the importance of considering psychological well-being in the assessment of adolescent chronic pain.

Poor sleep quality was also associated with higher odds of continuous chronic musculoskeletal pain. This finding is consistent with Andias and Silva (25), who reported associations between poorer sleep and disability among adolescents with chronic musculoskeletal pain. Nevertheless, longitudinal evidence remains inconsistent. A systematic review by Andreucci, Campbell and Dunn (26) found that sleep problems did not consistently predict the onset of musculoskeletal pain, although associations were identified in specific anatomical regions and subgroups. The present findings therefore support assessing sleep quality in adolescents with persistent pain, without establishing whether sleep disturbances precede or result from the painful condition.

Longer daily smartphone use remained associated with chronic musculoskeletal pain, with approximately 9% higher odds of pain per additional hour of use. This finding is consistent with a Colombian study reporting associations between mobile phone dependence and musculoskeletal pain, particularly neck pain (10). Similarly, a systematic review identified associations between screen exposure, sedentary behavior, and musculoskeletal pain in adolescents, although methodological heterogeneity limits definitive conclusions (27). Unlike studies investigating problematic smartphone use, the present study assessed usage duration rather than dependence, posture, or digital activities. Potential mechanisms involving sustained postures, reduced movement, or sleep disruption therefore remain hypothetical. Furthermore, researchers should consider smartphone use within adolescents’ social circumstances, including its role in education, communication, and access to information.

Although sex, depressive symptoms, self-rated health, and school bullying did not remain statistically significant after adjustment, these findings do not exclude their potential relevance to adolescent chronic pain. Previous research has identified associations between chronic pain, psychological distress and interpersonal factors, although findings vary according to pain characteristics and population (1, 7, 24). The absence of statistically significant adjusted associations in the present study should therefore be interpreted in relation to the model specification, sample characteristics and precision of the estimates.

The observed associations may reflect reciprocal and mutually reinforcing processes. Sleep disruption may increase pain sensitivity and negatively affect emotional functioning, while persistent pain may itself disturb sleep continuity. Recent evidence supports a bidirectional relationship between sleep disturbance and pain during adolescence, although the direction and magnitude of these effects may vary across individuals and over time (28). Similarly, anxiety may intensify pain-related distress and functional difficulties, whereas persistent pain may contribute to the development or maintenance of anxiety symptoms. A recent systematic review and meta-analysis showed that anxiety is highly prevalent among young people with chronic pain and may act both as a consequence of the pain experience and as a factor contributing to ongoing pain-related disability (24). These mechanisms are biologically and psychologically plausible but cannot be temporally ordered or tested as causal pathways using the present cross-sectional design.

From an equity perspective, these findings highlight the need to generate epidemiological evidence from socially disadvantaged populations that remain underrepresented in pediatric pain research. The coexistence of persistent pain, anxiety symptoms, poor sleep quality, and prolonged smartphone use supports the consideration of multidimensional assessment strategies in school and primary healthcare settings. Integrating pain assessment with psychological well-being, sleep and everyday behaviors may facilitate the identification of adolescents with complex health needs. However, further investigation is needed to assess the feasibility and effectiveness of these approaches in resource-constrained settings.

The study's strengths include territory-stratified school sampling, validated instruments, and the comprehensive assessment of biopsychosocial factors. Nevertheless, its cross-sectional design and reliance on self-reported data limit causal interpretation. The lack of detailed pain characteristics, healthcare-seeking behavior information, and individual socioeconomic indicators, along with recruitment from a single urban district, may limit the interpretation and generalizability of the findings. Future longitudinal, multicenter studies that incorporate these dimensions could strengthen the evidence base and inform more equitable, context-sensitive approaches to pediatric pain care.

## 5 Conclusion

Continuous chronic musculoskeletal pain was reported by nearly one-third of adolescents attending public schools in a socially vulnerable urban setting in north-eastern Brazil. In the adjusted analysis, poor sleep quality, anxiety symptoms, and longer daily smartphone use emerged as the main correlates of the outcome. These findings highlight the multidimensional profile of adolescent pain in an underrepresented Brazilian context and support the need for context-sensitive assessment in school and primary healthcare settings. Longitudinal studies incorporating individual socioeconomic indicators, detailed pain characteristics, and healthcare utilization are needed to clarify temporal pathways and potential inequities in pediatric pain care.

## Data Availability

The data that support the findings of this study are available from the corresponding author upon reasonable request.
